# Bub1 Is a Fission Yeast Kinetochore Scaffold Protein, and Is Sufficient to Recruit other Spindle Checkpoint Proteins to Ectopic Sites on Chromosomes

**DOI:** 10.1371/journal.pone.0001342

**Published:** 2007-12-19

**Authors:** Patricia E. Rischitor, Karen M. May, Kevin G. Hardwick

**Affiliations:** The Wellcome Trust Centre for Cell Biology, Institute of Cell Biology, University of Edinburgh, Edinburgh, United Kingdom; Duke University Medical Centre, United States of America

## Abstract

The spindle checkpoint delays anaphase onset until all chromosomes have attached in a bi-polar manner to the mitotic spindle. Mad and Bub proteins are recruited to unattached kinetochores, and generate diffusible anaphase inhibitors. Checkpoint models propose that Mad1 and Bub1 act as stable kinetochore-bound scaffolds, to enhance recruitment of Mad2 and Mad3/BubR1, but this remains untested for Bub1. Here, fission yeast FRAP experiments confirm that Bub1 stably binds kinetochores, and by tethering Bub1 to telomeres we demonstrate that it is sufficient to recruit anaphase inhibitors in a kinase-independent manner. We propose that the major checkpoint role for Bub1 is as a signalling scaffold.

## Introduction

Cells employ many mechanisms to ensure that their genomes are replicated and segregated with high fidelity every cell cycle [1]. Errors in chromosome segregation result in aneuploidy, which often leads to cell death and is strongly associated with cancer progression [2], [3]. During mitosis the spindle checkpoint monitors kinetochore-microtubule interactions, and only when all sister-chromatid pairs have achieved bi-orientation on the mitotic spindle is anaphase allowed to proceed. This checkpoint inhibits the activity of the anaphase-promoting complex (Cdc20-APC), preventing polyubiquitination and destruction of mitotic regulators such as securin and cyclin, and thereby delays anaphase onset [4], [5].

The molecular mechanism of action of the spindle checkpoint remains unclear, although several important findings have been made. First, a single unattached kinetochore is sufficient to activate the checkpoint [6]. Second, all of the checkpoint proteins are recruited to unattached kinetochores, as is their effector Cdc20 [7]–[10]. Third, a sub-set of checkpoint proteins, including Mad2 and BubR1/Mad3, form stable complexes with Cdc20 [11]–[13], which is the critical effector of the spindle checkpoint [14], [15]. Such checkpoint protein complexes are sufficient to inhibit Cdc20-APC activity *in vitro* [13], [16], [17].

Here we focus on the mechanism of recruitment of checkpoint proteins to kinetochores, and their exchange dynamics once recruited. Several fluorescence recovery after photo-bleaching (FRAP) studies have described the dynamics of spindle checkpoint proteins and Cdc20 in vertebrate cells [7]–[10]. These employed either transient transfection of GFP tagged checkpoint constructs, or the production of stable cell lines expressing fusion proteins, and in all cases the cell lines also contained the endogenous wild-type checkpoint protein. This is a major limitation of these studies as it is possible that the GFP fusion proteins do not reflect the behaviour of the wild-type protein. In addition to the possibility that the GFP tag perturbs function, the endogenous protein could out-compete the GFP fusion protein for binding sites on chromosomes. If these were rare and/or stable binding sites, this would have a major influence on the dynamic parameters measured. Vink et al have reconstituted dynamic aspects of Mad2 behaviour *in vitro*, using Mad1/Mad2 “scaffolds” coupled to beads [18]. These FRAP studies demonstrate that Mad2 behaviour is rather complex: there is a stable kinetochore-bound pool of Mad2, tightly bound to Mad1, and a dynamic Mad2 pool that rapidly exchanges. In kinetochore FRAP experiments, 50–90% of Mad2 recovers after the first bleach (the dynamic pool) with a half-time of 6–25 seconds (see [18] for Tables comparing different kinetic analyses). This dynamic exchange of Mad2 molecules is thought to be critical for Cdc20 interaction and inhibition [19], [20]. As yet, no *in vitro* work has been reported for BubR1/Mad3, Bub3 or Bub1 dynamics.

In fission yeast, Bub1p is necessary for the efficient recruitment of Bub3p and Mad3p to kinetochores, and their targeting is independent of Mad1p and Mad2p [21]. Mutations within the highly conserved N-terminal domain of Bub1p dramatically reduced its own kinetochore targeting, and that of Bub3p, and practically abolished Mad3p kinetochore enrichment [21], [22]. Thus both Bub1p and Mad1p are thought to be kinetochore-based checkpoint scaffolds. Here we demonstrate that Bub1p is a relatively stable component of mitotic kinetochores in fission yeast, and that when ectopically targeted to telomeres it is sufficient to recruit both Bub3p and Mad3p to these ectopic sites on chromosomes.

## Results and Discussion

### Fission yeast Bub1p is stably associated with mitotic kinetochores

**Figure 1:**
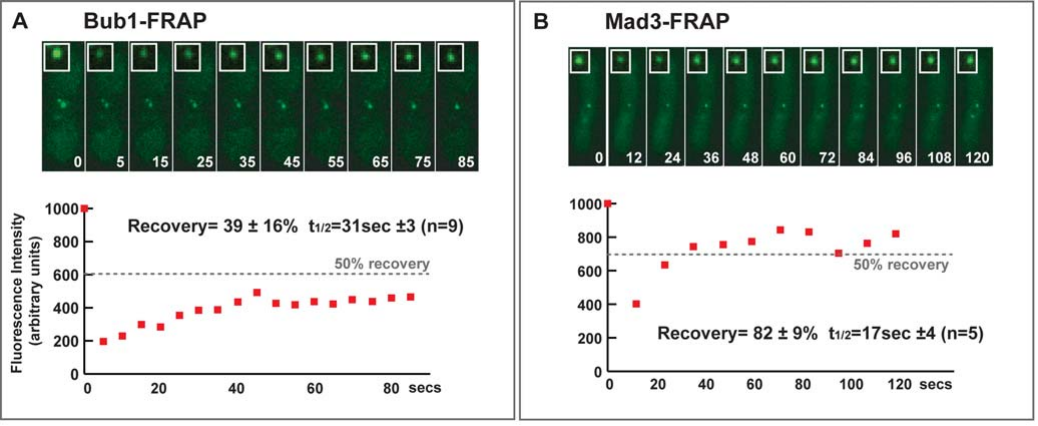
Bub1p is a relatively stable component of fission yeast kinetochores, whereas the bulk of Mad3p rapidly exchanges. (A) Bub1-GFP fluorescence recovery after photo-bleaching (FRAP): *nda3* cells expressing Bub1-GFP were arrested in mitosis at 18°C and treated with anti-microtubule drugs (25 µg/ml carbendazim) to ensure the arrest was maintained. Specific GFP kinetochore signals were then photobleached with a laser, and images captured at intervals throughout the recovery period. The % fluorescence recovery and half-times indicated are the average of nine experiments. The recovery curve shown is representative, and the dashed line indicates the 50% post-bleach recovery level. (B) Mad3-GFP fluorescence recovery after photo-bleaching (FRAP): *nda3* cells expressing Mad3-GFP were arrested in mitosis at 18°C and treated with anti-microtubule drugs (25 µg/ml carbendazim) to ensure the arrest was maintained. Specific GFP kinetochore signals were then photobleached with a laser, and images captured at intervals throughout the recovery period. The % fluorescence recovery and half-times indicated are the average of 5 experiments.

As mentioned above, there are a number of caveats with the published FRAP studies on the intracellular dynamics of spindle checkpoint proteins. Vertebrate studies have argued that Bub1-GFP is a relatively stable kinetochore component. Less than 20% recovery was observed after bleaching cell lines stably expressing YFP-Bub1 [10], and in cells transiently transfected with GFP-Bub1 56% recovered with a t_1/2 _of ∼30 seconds [8]. The fission yeast wild-type *bub1+* gene has been C-terminally tagged with GFP, such that it is expressed from its own promoter at the endogenous locus, and a range of checkpoint and chromosome segregation assays demonstrate that it is fully functional [21]–[23]. To analyse the dynamics of Bub1-GFP at unattached kinetochores, we used a cold-sensitive tubulin mutant (*nda3*). These cells arrest early in mitosis, with no microtubules, unattached kinetochores and hyper-condensed chromosomes [24]. In addition, we treated these *nda3* cells with anti-microtubule drugs (25 µg/ml carbendazim) to ensure the arrest was maintained. We carried out FRAP experiments and a representative example is shown (Fig. 1A). Analysis of the recovery profiles (see Supplementary material) showed that Bub1-GFP displayed 39 (±16) % recovery, and that the dynamic pool recovered with a half-time of ∼31 (+/−3) seconds (n = 9). This value is mid-way between the two published recovery profiles for vertebrate Bub1.

### Fission yeast Mad3p exchanges rapidly at mitotic kinetochores

FRAP studies of vertebrate BubR1, which is the Mad3 homologue, have shown that it is one of the most dynamic checkpoint components [8]. To determine whether this was also true in fission yeast, and as a direct comparison for Bub1p dynamics, we carried out FRAP experiments with Mad3-GFP. The fission yeast wild-type *mad3+* gene has been C-terminally tagged with GFP, such that it is expressed from its own promoter at the endogenous locus, and a range of checkpoint and chromosome segregation assays demonstrate that it is fully functional [12], [21], [22]. Mad3-GFP is recruited to kinetochores early in mitosis, and the signal becomes greatly reduced during anaphase [12]. To analyse the dynamics of Mad3-GFP at unattached kinetochores, we used a cold-sensitive tubulin mutant (*nda3*) and arrested it in mitosis at 18°C for 6 hours. Here we observed 82 (+/−9) % recovery of Mad3-GFP, with a recovery half-time of 17 (+/−4) seconds (Fig. 1B). We conclude that Mad3-GFP is being rapidly recruited to and then released from kinetochores early in mitosis. These Mad3p kinetics are similar to those previously reported for vertebrate BubR1 [8].

We conclude from these experiments that fission yeast checkpoint proteins display similar *in vivo* dynamics to those previously measured in vertebrate cell lines, and that Bub1p is a relatively stable component of fission yeast kinetochores. These are important confirmations of vertebrate checkpoint FRAP studies, and once again validate fission yeast kinetochores and checkpoint proteins as excellent models of their human equivalents. The above experiments employ arrested *nda3* cells and it was recently shown that some kinetochores in these cells remain associated with spindle pole bodies [25]. Thus it is possible that some of the kinetochores we analysed by FRAP were attached to microtubules and that others were unattached. This may account for some of the variation observed in Bub1p and Mad3p behaviour between experiments, but further analysis is required to address this issue more thoroughly.

### Bub1p is sufficient to recruit both Bub3p and Mad3p to ectopic sites on chromosomes

**Figure 2:**
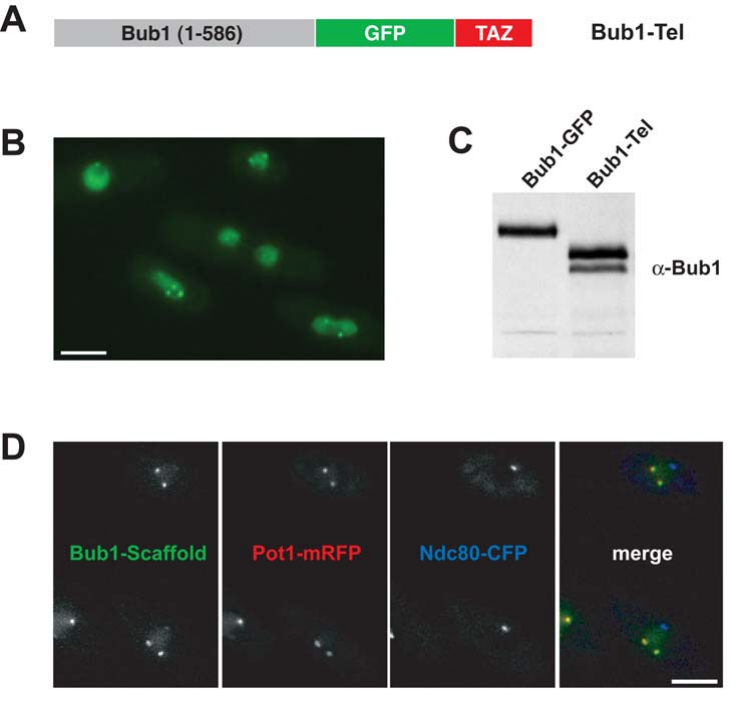
Bub1-(1-586)-GFP-Taz1Myb is targeted to telomeres. (A) A model of the Bub1-GFP-Taz scaffold protein (Bub1-Tel). (B) Field of *bub1Δ* cells expressing Bub1-Tel. (C) Anti-Bub1p immunoblot of extracts from strains expressing Bub1-GFP or Bub1-Tel. (D) Bub1-Tel co-localises with telomeres (Pot1-mRFP) and not kinetochores (Ndc80-CFP) in interphase cells. In mitotic cells the scaffold is recruited to both telomeres and kinetochores (data not shown). Scale bar is 5 microns.

From loss of function studies we have argued that Bub1p might act as a scaffold to recruit other checkpoint components [21], in much the same way as proposed for Mad1 in the recruitment of Mad2 to kinetochores. To directly test the model that Bub1p is a checkpoint scaffold, we chose to target Bub1p to fission yeast telomeres. The N-terminal 586 residues of Bub1p are known to be sufficient for checkpoint arrest [21], so we fused these to GFP and a telomere targeting domain. The Myb DNA binding domain of the fission yeast telomere-binding protein Taz1p, has been shown to be sufficient for recruitment of other factors to telomeres [26]. We made a Bub1-GFP-Taz(Myb) fusion protein (Fig. 2A), hereafter referred to as Bub1-Tel, and expressed it in both wild-type and *bub1Δ* strains. Multiple, distinct GFP foci were observed in interphase cells, which were very reminiscent of telomeres (Fig. 2B). Immunoblots show that Bub1-Tel was expressed as a stable fusion protein (Fig. 2C). To confirm its telomeric localisation, we crossed the Bub1-Tel into strains expressing fluorescent kinetochore (Ndc80-CFP) and telomere (Pot1-mRFP) markers. Whilst in some cells, weak staining was observed at centromeres, the majority of the Bub1-Tel foci co-localised with the Pot1 telomere marker, indicating that this scaffold protein had been successfully targeted to telomeres (Fig. 2D). In mitotic cells the Bub1-Tel localised to both telomeres and centromeres (data not shown).

**Figure 3:**
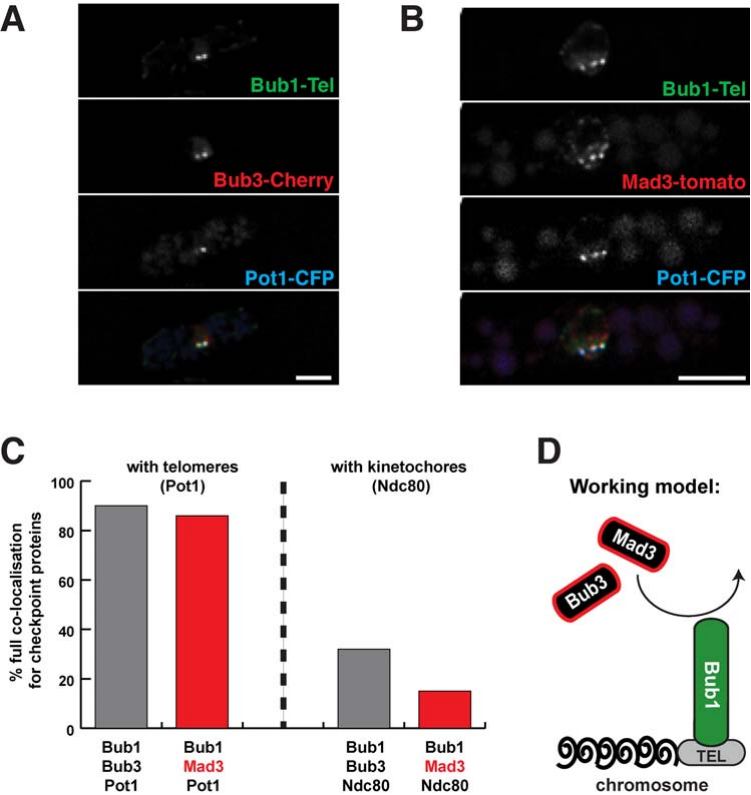
Bub1-Tel is sufficient to recruit both Bub3p and Mad3p. (A) Bub1-Tel recruits and co-localises with Bub3-mCherry at telomeres. (B) Bub1-Tel recruits and co-localises with Mad3-tdTomato at telomeres. Scale bars are 5 µm. (C) Quantitation of the co-localisation observed between checkpoint proteins and telomeres (Pot1), or kinetochores (Ndc80). Full co-localisation was scored when all of the telomere (or kinetochore) foci observed in a given cell co-localised with the Bub1-Tel *and* either Bub3p (grey columns) or Mad3p (red columns). See supplementary data (Tables S1, S2, S3 and S4) for further details. (D) Speculative model of Bub1 scaffold action at a telomere (TEL). Note, due to low signal intensity, we have not yet demonstrated that Bub3p and Mad3p exchange rapidly at the telomeres.

To test whether this Bub1-scaffold was sufficient to recruit Bub3p and Mad3p to telomeres we crossed in either Mad3-tdTomato or Bub3-mCherry. The Mad3 and Bub3 fusion proteins co-localised very well with the telomere marker (Pot1-CFP) and the Bub1-Tel (GFP) (Fig. 3 and Supplementary Tables S1, S2, S3 and S4). This simple experiment demonstrates that targeting of Bub1p to an ectopic site on a chromosome, is sufficient to recruit other checkpoint proteins to that site.

Here we have tested critical aspects of the scaffolding model for Bub1 checkpoint function. Bub1p is relatively stably associated with kinetochores, and is sufficient to recruit other checkpoint proteins, and therefore displays the two key characteristics of a signalling scaffold. Note, our scaffold lacks the C-terminal kinase domain, confirming that this is not necessary for the recruitment of Bub3p and Mad3p. This ectopic targeting of Bub1p, Bub3p and Mad3p to telomeres had no consequence on cell cycle progression. Unfortunately the levels of Bub3p and Mad3p recruited to the telomeres were not sufficient to carry out detailed FRAP studies. These are important experiments for the future, and we will extend this approach by co-targeting of a Mad1p scaffold, and test whether the concerted action of Mad1p and Bub1p is sufficient for generation of “wait anaphase” signals.

It is not clear why all the checkpoint proteins are recruited to kinetochores. We speculate that certain checkpoint proteins, and perhaps the Cdc20 effector, not only undergo structural conformational change [19], but also receive post-translational modification when associated with kinetochores. We do not think that such modifications are carried out by Bub1 kinase itself, as its kinase domain is not necessary for checkpoint arrest in yeast, although it may play a role in humans [27]. Mps1 [28], Aurora [29], CDK [30] and/or MAP kinases [31] could all have important roles in the phosphorylation and activation of anaphase inhibitors, or the sensitisation of their Cdc20-APC target [32]. By building more complex scaffolds that also recruit such protein kinases we can dissect these checkpoint signaling events.

## Materials and Methods

See Table 1 for yeast strains.

**Table 1:** Yeast strains

Yeast strain	Genotype
KMP134	*Bub1-GFP::his3 cut12CFP::G418 nda3-KM311*
DM076	*Mad3-GFP::his3 nda3-KM311*
SPR121	*h^− ^bub1^1–586^-GFP-taz1myb::leu1 bub1Δ::ura4 lys1*
SPR170	*h^− ^bub1^1–586^-GFP-taz1myb::leu1 bub3-mCherry::CLONAT ndc80-CFP::G418 bub1Δ::ura4 lys1*
SPR177	*h^− ^bub1^1–586^-GFP-taz1myb::leu1 mad3-mCherry::CLONAT ndc80-CFP::G418 bub1Δ::ura4 lys1*
SPR159	*h^− ^bub1^1-586^-GFP-taz1myb::leu1 pot1-mRFP::G418 ndc80-CFP::G418 bub1Δ::ura4 lys1*
SPR198	*h^+ ^bub1^1–586^-GFP-taz1myb::leu1 pot1-CFP::G418 bub3-mCherry::CLONAT bub1Δ::ura4 lys1*
SPR201	*h^+ ^bub1^1–586^-GFP-taz1myb::leu1 pot1-CFP::G418 mad3-mTomato::CLONAT bub1Δ::ura4 lys1*
SPR208	*h^−^ pot1-mRFP::G418 ndc80-CFP::G418*
SPR212	*h^− ^bub1^1–586^ Δ28-160-GFP-taz1myb::leu1 ndc80-CFP::G418 bub1Δ::ura4 lys1*

### Construction of the Bub1-GFP-Taz1Myb scaffold

Bub1^1–586^-GFP-Taz1Myb was constructed as follows. The *bub1* promoter (500bp upstream of the *bub1* ATG) plus a 1758 bp fragment of *bub1+* encoding the first 586 residues was amplified from *S. pombe* genomic DNA with a *Not*I added at the 5′ end and *Sma*I at the 3′ end of the fragment. This was inserted into the MCS of the plasmid pJK148 [33]. Here primers used were: Bub1NotFW–CGTAGCGGCCGCGATGATGCATTTGATGTTTAAG and Bub1SmaREV–CGTACCCGGGCGTGGCTACCGGATTAC. The DNA fragment containing the C-terminal 167 amino acid residues of Taz1, fused to the Myb DNA binding domain (Taz1Myb) [26], was PCR amplified from plasmid pYC798 (kind gift from Y. Hiraoka), with *Pst*I added at the 5′ end and *Sal*I at the 3′ end of the fragment and ligated into the same unique sites of the plasmid, in-frame to the 3′ end of the Bub1^1–586^ fragment. Primers used were: GFPTaz1PstIFW–CGATCTGCAGATGAGTAAAGGAGAAGAAC and GFPTaz1SalIRV–GCCGTCGACTTAAGATTGATAATTAACAAG. The resulting plasmid was integrated into the chromosome at the *leu1* gene locus in cells disrupted for the *bub1+* gene.

### Tagging strategies

The mCherry and tdTomato fusion constructs with Bub3 and Mad3 respectively were made using the PCR-based gene targeting method [34]. Plasmids containing mCherry and tdTomato were kindly provided by Ken Sawin [35], [36].

### Immunoblotting

Preparation of yeast cell extracts, SDS-PAGE and immunoblotting were carried out as previously described [37].

### Imaging

Live-cell imaging was typically performed in minimal media, on a pad of 1% agarose. Some GFP, mCherry and tdTomato imaging (Figure 2c and d) was also performed after brief methanol fixation (30 to 60 s). All microscopy was carried out using an Intelligent Imaging Innovations (3i) Marianas microscope (Zeiss Axiovert 200M), using a 100x 1.3NA objective lens, CoolSnap CCD and Slidebook software (3i, Boulder). The images shown in Fig. 2 are maximum intensity projections of Z stacks.

This system was also used for FRAP: cells were mounted on a pad of 1% agarose and PMG media with 25 µg/ml carbendazim (CBZ) and sealed with VALAP (Vaseline/Lanolin/Paraffin). Photobleaching was carried out using a 514nm Nitrogen dye laser system (Photonic Instruments). Images were captured and analysed using Slidebook software (3i, Boulder). To calculate fluorescence recovery, images were captured with 200ms exposure at 2x2 binning at various time intervals post bleaching. The kinetics of FRAP were analysed as described [38]. Briefly, fluorescence intensity was determined using the integrated intensity of a 5x5 pixel box. To correct for background the mean intensity of 3 regions was subtracted from the intensity of the bleached region over the kinetochore at each time point. The proportion of Bub1-GFP and Mad3-GFP remaining unbleached was calculated from pre and post bleach whole cell fluorescence intensities and used to correct for the pool irreversibly bleached during the experiment.

## Supporting Information

Table S1 Analysis of co-localisation between Bub1-TEL, Mad3, and kinetochores (Ndc80).
(0.05 MB PDF)

Table S2 Analysis of co-localisation between Bub1-Tel, Bub3 and kinetochores (Ndc80).
(0.05 MB PDF)

Table S3 Analysis of co-localisation between Bub1-Tel, Mad3 and telomeres (Pot1).
(0.04 MB PDF)

Table S4 Analysis of co-localisation between Bub1-Tel, Bub3 and telomeres (Pot1).
(0.05 MB PDF)
