# Supplementary material for: Bub1 Is a Fission Yeast Kinetochore Scaffold Protein, and Is Sufficient to Recruit other Spindle Checkpoint Proteins to Ectopic Sites on Chromosomes
Source: PLoS One. 2007 Dec 19;2(12):e1342. doi: 10.1371/journal.pone.0001342 (PMC2147072; doi:10.1371/journal.pone.0001342)
Supplement: Table S1 — Analysis of co-localisation between Bub1-TEL, Mad3, and kinetochores (Ndc80). (0.05 MB PDF) [file pone.0001342.s001.pdf]

Supplementary Table S1

Number of Bub1-Tel/Mad3 dots co-localising with Ndc80 (KT, kinetochore) (shown as a %, 50 cells counted in total)

1B1/<br>1M3/<br>1KT	2B1/<br>2M3/<br>1KT	3B1/<br>3M3/<br>1KT	4B1/<br>4M3/<br>1KT	2B1/<br>2M3/<br>NO KT	3B1/<br>3M3/<br>NO KT	5B1/<br>5M3/<br>NO KT
15	37	29	11	4	2	2

e.g. 3B1/3M3/1KT means that 3 Bub1 foci were observed in a cell, and all co-localised with Mad3tdTomato, but that only one co-localised with Ndc80. NO KT means that none of the Bub1 or Mad3 foci co-localised with Ndc80.

The majority of cells contain multiple co-localising foci for Bub1-Tel and Mad3, and only one (or none) of these co-localised with Ndc80 (kinetochores).
