# Supplementary material for: Bub1 Is a Fission Yeast Kinetochore Scaffold Protein, and Is Sufficient to Recruit other Spindle Checkpoint Proteins to Ectopic Sites on Chromosomes
Source: PLoS One. 2007 Dec 19;2(12):e1342. doi: 10.1371/journal.pone.0001342 (PMC2147072; doi:10.1371/journal.pone.0001342)
Supplement: Table S2 — Analysis of co-localisation between Bub1-Tel, Bub3 and kinetochores (Ndc80). (0.05 MB PDF) [file pone.0001342.s002.pdf]

Supplementary Table S2

Number of Bub1-Tel/Bub3 dots co-localising with Ndc80 (KT, kinetochore) (shown as a %, 50 cells counted in total)

1B1/ 1B3/ 1KT	2B1/ 2B3/ 1KT	3B1/ 3B3/ 1KT	4B1/ 4B3/ 1KT	1B1/ 1B3/ NO KT	2B1/ 2B3/ NO KT	3B1/ 3B3/ NO KT	5B1/ 5B3/ NO KT
32	16	16	16	8	4	6	2

The majority of cells contain multiple co-localising foci for Bub1-Tel and Bub3, and only one (or none) of these co-localised with Ndc80 (kinetochores).
