# Supplementary material for: Bub1 Is a Fission Yeast Kinetochore Scaffold Protein, and Is Sufficient to Recruit other Spindle Checkpoint Proteins to Ectopic Sites on Chromosomes
Source: PLoS One. 2007 Dec 19;2(12):e1342. doi: 10.1371/journal.pone.0001342 (PMC2147072; doi:10.1371/journal.pone.0001342)
Supplement: Table S3 — Analysis of co-localisation between Bub1-Tel, Mad3 and telomeres (Pot1). (0.04 MB PDF) [file pone.0001342.s003.pdf]

Supplementary Table S3

Number of Bub1/Mad3 dots co-localising with Pot1 (T, telomere) (shown as a %, 28 cells counted in total)

1B1/<br>1M3/<br>1T	2B1/<br>2M3/<br>2T	3B1/<br>3M3/<br>1T	3B1/<br>3M3/<br>3T	4B1/<br>4M3/<br>4T	3B1/1T<br>2M3/1T	4B1/4T<br>2M3/2T	2B1/1T<br>1M3/1T
58	7	3.5	14	7	3.5	3.5	3.5

Almost all cells contain at least one co-localising foci for Bub1-Tel and Mad3 that also co-localises with Pot1 (telomeres).
