# Supplementary material for: Bub1 Is a Fission Yeast Kinetochore Scaffold Protein, and Is Sufficient to Recruit other Spindle Checkpoint Proteins to Ectopic Sites on Chromosomes
Source: PLoS One. 2007 Dec 19;2(12):e1342. doi: 10.1371/journal.pone.0001342 (PMC2147072; doi:10.1371/journal.pone.0001342)
Supplement: Table S4 — Analysis of co-localisation between Bub1-Tel, Bub3 and telomeres (Pot1). (0.05 MB PDF) [file pone.0001342.s004.pdf]

Supplementary Table S4

Number of Bub1/Bub3 dots co-localising with Pot1 (T, telomere) (shown as a %, 20 cells counted in total)

1B1/<br>1B3/<br>1T	2B1/<br>2B3/<br>1T	2B1/<br>2B3/<br>2T	3B1/<br>3B3/<br>3T	4B1/<br>4B3/<br>4T	5B1/<br>5B3/<br>5T	3B1/1T<br>4B3/4T
5	5	20	30	25	10	5

Almost all cells contain at least one (and typically several) co-localising foci for Bub1-Tel and Bub3 that also co-localises with Pot1 (telomeres).
